# Evaluation of Metabolism of a Defined Pesticide Mixture through Multiple In Vitro Liver Models

**DOI:** 10.3390/toxics10100566

**Published:** 2022-09-27

**Authors:** Alan Valdiviezo, Yuki Kato, Erin S. Baker, Weihsueh A. Chiu, Ivan Rusyn

**Affiliations:** 1 Interdisciplinary Faculty of Toxicology, College of Veterinary Medicine and Biomedical Sciences, Texas A&M University, College Station, TX 77843, USA; 2 Department of Veterinary Physiology and Pharmacology, College of Veterinary Medicine and Biomedical Sciences, Texas A&M University, College Station, TX 77843, USA; 3 Laboratory for Drug Discovery and Development, Shionogi Pharmaceutical Research Center, Shionogi & Co., Ltd., Osaka 561-0825, Japan; 4 Department of Chemistry, North Carolina State University, Raleigh, NC 27695, USA

**Keywords:** toxicokinetics, chemical mixtures, defined mixtures, human health risk assessment, nontargeted analyses, microphysiological systems

## Abstract

The evaluation of exposure to multiple contaminants in a mixture presents a number of challenges. For example, the characterization of chemical metabolism in a mixture setting remains a research area with critical knowledge gaps. Studies of chemical metabolism typically utilize suspension cultures of primary human hepatocytes; however, this model is not suitable for studies of more extended exposures and donor-to-donor variability in a metabolic capacity is unavoidable. To address this issue, we utilized several in vitro models based on human-induced pluripotent stem cell (iPSC)-derived hepatocytes (iHep) to characterize the metabolism of an equimolar (1 or 5 µM) mixture of 20 pesticides. We used iHep suspensions and 2D sandwich cultures, and a microphysiological system OrganoPlate^®^ 2-lane 96 (Mimetas^TM^) that also included endothelial cells and THP-1 cell-derived macrophages. When cell culture media were evaluated using gas and liquid chromatography coupled to tandem mass spectrometry methods, we found that the parent molecule concentrations diminished, consistent with metabolic activity. This effect was most pronounced in iHep suspensions with a 1 µM mixture, and was lowest in OrganoPlate^®^ 2-lane 96 for both mixtures. Additionally, we used ion mobility spectrometry–mass spectrometry (IMS-MS) to screen for metabolite formation in these cultures. These analyses revealed the presence of five primary metabolites that allowed for a more comprehensive evaluation of chemical metabolism in vitro. These findings suggest that iHep-based suspension assays maintain higher metabolic activity compared to 2D sandwich and OrganoPlate^®^ 2-lane 96 model. Moreover, this study illustrates that IMS-MS can characterize in vitro metabolite formation following exposure to mixtures of environmental contaminants.

## 1. Introduction

Environmental chemicals typically have low solubility in aqueous systems and require biotransformation to metabolites that are less lipophilic and more readily eliminated. Most chemical metabolism occurs in two phases [1]. Phase I reactions (e.g., oxidation, reduction, or hydrolysis) serve to convert lipophilic compounds into more polar molecules by adding or revealing a polar functional group [2,3,4]. Phase II reactions involve the conjugation of metabolites via glucuronidation, sulfation, methylation, or acetylation to create compounds that are much more soluble and, therefore, more easily eliminated [5,6,7]. Generally, metabolism leads to the detoxification of xenobiotics by creating inactive metabolites; however, intermediate products created during metabolism can be toxic and reactive [6,8,9]. Therefore, it is critical to understand and characterize the metabolism of xenobiotics to better predict potential toxicity.

Traditional toxicity testing relies on evaluating chemicals on an individual basis. However, humans are usually exposed to numerous chemicals that exist as mixtures in real life [10,11,12]. Exposure to chemical mixtures constitutes a major challenge for risk assessment. Understanding the metabolism of compounds in a mixture setting remains largely unexplored. Studies to evaluate the cumulative toxicity of mixtures in animal models are costly and time consuming [13,14,15,16]. Additionally, animal data may not accurately reflect human biokinetics of xenobiotics, which further hinders extrapolation to humans. Therefore, alternative approaches for the assessment of xenobiotic metabolism associated with chemical mixtures are needed to better characterize potential hazards to human and environmental health.

In vitro methods to study xenobiotic metabolism in liver-derived cells can address the limitations associated with in vivo testing by enabling high-throughput screening at much lower costs. Traditional assays such as hepatocyte suspensions and 2D cultures have been widely used for rapid screening and characterization of xenobiotic metabolism [8,17,18]; however, these approaches have limitations. Due to the time-dependent loss of cell function and viability in suspension assays, the metabolism of low-turnover compounds tends to be underestimated [17]. In addition, monolayer cultures tend to underestimate the clearance of high-turnover compounds, likely due to an uptake rate limitation [19]. New approach methods including multicell-based models such as microphysiological systems of the liver can potentially overcome the limitations associated with cell suspensions and monolayer cultures [20,21]. Microphysiological systems enable the understanding of complex biological systems and facilitate chemical screening for toxicity to human health [22,23,24]. For example, the OrganoPlate^®^ 2-lane 96 liver model was used for hepatotoxicity screening [23,25]; however, xenobiotic metabolism studies in this device remain largely unexplored. Therefore, identifying the utility of the OrganoPlate^®^ 2-lane 96 model for the characterization of biokinetics merits further attention.

Evaluating the metabolism of xenobiotics through in vitro systems is critical for characterizing toxicokinetics; however, determining the formation of metabolites is equally important, especially when extrapolating results to in vivo predictions. Traditional methods to assess in vitro metabolite formation involve targeted analytical methods [26,27,28]. However, targeted analyses may not detect the presence of metabolites due to sensitivity issues. Therefore, a more comprehensive approach is needed to detect the presence of potentially toxic metabolites. Nontargeted analyses through high-resolution mass spectrometry enable the rapid characterization of hundreds to thousands of compounds in a given environmental or biological sample [29]. This approach has been previously shown to provide a more comprehensive compositional characterization of environmental contaminant presence in the environment compared to targeted methods [30]. Recent advances in analytical tools including ion mobility spectrometry–mass spectrometry (IMS-MS) facilitate nontargeted analyses in a rapid manner and have shown to be an appealing technique for nontargeted metabolomics [31,32]. Furthermore, this technique can potentially reveal the presence of metabolites formed from in vitro studies.

**Figure 1:**
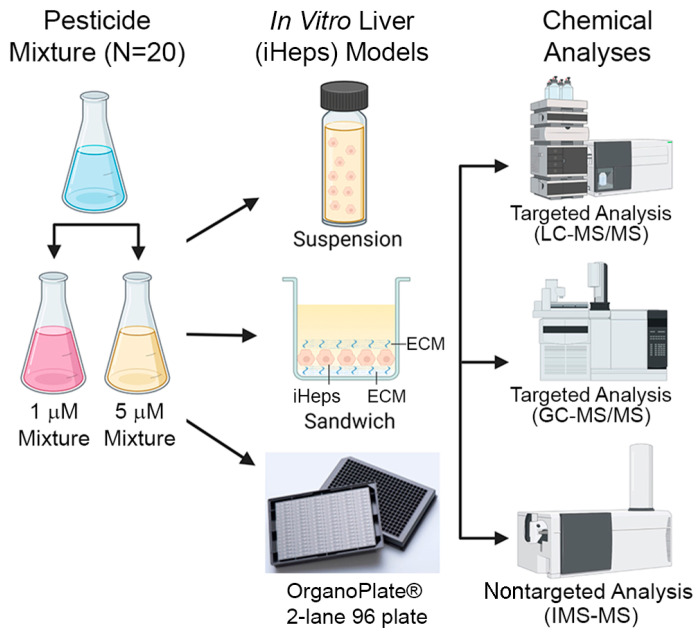
Experimental design. Schematic diagram describing the chemical mixture tested across different in vitro liver models (suspension, 2D sandwich, and OrganoPlate^®^ 2-lane 96) and types of chemical analyses used in this study.

In this study, we utilized three in vitro liver models to evaluate the metabolism of pesticides in mixtures: suspension, 2D sandwich, and OrganoPlate^®^ 2-lane 96 (Figure 1). Twenty pesticides were used to create equimolar mixtures (1 or 5 µM, each chemical). Induced pluripotent stem cell-derived hepatocytes (iHep) were used in each in vitro model. In addition to iHep suspensions and 2D cultures, the OrganoPlate^®^ 2-lane 96 was used and included non-parenchymal cells (macrophages and endothelial). Following chemical exposure, we measured albumin production and cell damage. Next, we determined the clearance rate for each compound with traditional mass spectrometry methods and IMS-MS. Additionally, we screened for metabolite formation using IMS-MS. The results of this study are informative for the assessment of metabolic capacity between traditional in vitro metabolism models and a novel microphysiological system. Moreover, this study further illustrates the utility of IMS-MS for rapid screening of xenobiotic metabolites following exposure to mixtures of environmental chemicals.

## 2. Experimental Section

### 2.1. Chemicals

Thirty-five compounds were used in this study as analytes or standards (Table 1) and were purchased from Sigma-Aldrich (St Louis, MO, USA), Chem Service (West Chester, PA, USA), or Toronto Research Chemicals (Toronto, ON, Canada). Methanol (Cat No.: 646377), acetonitrile (Cat No.: 34998), pentane (Cat No.: 34956), diethyl ether (Cat No.: 309966), and distilled water with 0.1% formic acid (Cat No.: 576913) were purchased from Sigma-Aldrich (St. Louis, MO, USA).

### 2.2. Cell Culture Reagents and Materials

Human-induced pluripotent stem cell-derived hepatocytes (iCell Hepatocytes 2.0, abbreviated herein as iHep) were purchased from FujiFilm-Cellular Dynamics International (Cat No.: C1023, lot#103934, Santa Ana, CA, USA). iHep plating media consisted of DMEM/F12 (Cat No.: 21041025, ThermoFisher, Waltham, MA, USA) supplemented with 2% B-27 supplement (Cat No.: 17504044, ThermoFisher), 100 nM dexamethasone (Cat No.: 265005, Millipore Sigma), 25 µg/mL gentamicin (15710072, ThermoFisher), and 20 ng/mL oncostatin M (Cat No.: 295-OM-010, R&D Systems, Minneapolis, MN, USA). It was used for pre-differentiation of iHep according to the manufacturer’s protocol. iHep maintenance media consisted of DMEM/F12, 2% B-27, 100 nM dexamethasone, and 25 μg/mL gentamicin; it was used for cell culture in the OrganoPlate^®^ 2-lane 96 and in the 384-well plates.

The microfluidic tissue chips used in this study, OrganoPlate^®^ 2-lane 96, were purchased from Mimetas (Leiden, Netherlands). Each device on this 96-well platform contains one gel channel and one perfusion channel. This configuration enables the culture of a perfused tubule adjacent to the extracellular matrix (ECM) of choice without a membrane [23]. Black-walled, clear-bottom, tissue culture-treated 96-well (Cat No.: 3603, Corning, Corning, NY, USA) and 384-well plates (Cat No.: 3765, Corning) were used for 2D cell culture experiments.

### 2.3. Preparation of Chemical Mixtures

The pesticides tested in this study were chosen from the ATSDR Substance Priority List, which contains compounds that are commonly detected at Superfund sites and are known to be hazardous to human health [33]. Molar-equivalent mixtures were created by combining all 20 pesticides and diluting them to a final concentration of 1 or 5 µM each. The final amount of dimethyl sulfoxide (DMSO) in either equimolar mixture (1 or 5 µM) did not exceed 0.5% *v*/*v*.

### 2.4. iHep Suspension Assays

Suspension assays using iHep were performed as previously detailed [34] with slight modification. In brief, pre-differentiated iHep were suspended in iHep maintenance media and adjusted to the cell concentration of 1 × 10^6^ cells/mL. A portion of the cell working stock was heated at 95 °C for five minutes to serve as negative control. Five hundred microliters of the chemical stock (20 chemicals, 2 or 10 µM each) were spiked in 500 µL of the cell working stock or heat-inactivated cell control to a final cell density of 5 × 10^5^ cells/mL. Fifty microliters were removed subsequently at 0, 60, 120, and 240 min to individual 1.5 mL Eppendorf tubes for further sample extraction detailed below. Each experimental condition was replicated three times.

### 2.5. iHep Culture in OrganoPlate^®^ 2-Lane 96, 384-Well Plates, and Chemical Treatments

The day when cells were seeded into OrganoPlate^®^ 2-lane 96 and 384-well plates was defined as Day 0. iHep were cultured in OrganoPlate^®^ 2-lane 96 using the protocol described elsewhere [23]. Briefly, thawed iHep were seeded at a density 2.5 × 10^6^ cells/well on a 6-well plate pre-coated with type 1 collagen (657950-005, Greiner Bio-One North America, Monroe, NC, USA) in iHep plating media. The cells were cultured for 4 h and unattached cells were removed when the media were replaced with fresh iHep plating media. The cells were differentiated for 5 days with daily changes in plating media. The differentiated iHep clusters were collected by centrifugation (200× *g*, 3 min) and resuspended into 3.33 mg/mL collagen (Cultrex 3-D Culture Matrix Rat Collagen-I, 3447-020-01, R&D Systems; 5 mg/mL type 1 collagen, 1 M HEPES, 37 g/L sodium bicarbonate at a ratio of 4:1:1, respectively) at a density of approximately 8.0 × 10^6^ cells/mL. The iHep/collagen suspension (2.5 μL/device) was gently injected into the inlet of the gel channel of each of the 96 devices on the plate using multi-channel electronic pipettor. After that, the whole plate was placed at 37 °C, 5% CO_2_ for 15 min to allow polymerization of the type 1 collagen. For negative control, iHep were heated at 95 °C for 5 min prior to loading into OrganoPlate 2-lane 96.

THP-1 monocytes and HMEC-1 endothelial cells were obtained from ATCC (Manassas, VA, USA). THP-1 monocytes were cultured in RPMI (Cat. No: 30-2001, ATCC) with 10% fetal bovine serum (Cat. No: 30-2020, ATCC) and 50 nM 2-mercaptoethanol (Cat. No: M3148, Millipore Sigma, Burlington, MA, USA). THP-1 monocytes were differentiated [35] into adherent macrophages via treatment with 250 nM phorbol 12-myristate-13-acetate (Cat. No: 356150050, ThermoFisher) for 48 h prior to seeding into OrganoPlate^®^ 2-lane 96 or multi-well plates. HMEC-1s were cultured in Molecular, Cellular, and Developmental Biology (MCDB) 131 medium (Cat. No: 10372019, ThermoFisher) with 10% fetal bovine serum (Cat. No: 30-2020, ATCC), 2 mM L-glutamine (Cat. No: 30-2214, ATCC), 100 units/mL penicillin-streptomycin (Cat. No: P0781, Millipore Sigma), 1 µg/mL hydrocortisone (Cat. No: H0888, Millipore Sigma), and 10 ng/mL epidermal growth factor recombinant human protein (Cat. No: PHG0314, ThermoFisher). A mixture of HMEC-1s at 40 × 10^6^ cells/mL and differentiated THP-1s at 3 × 10^6^ cells/mL was prepared in iHep maintenance media. After that, 2.5 μL HMEC-1/THP-1 cell suspension was injected into the inlets of the perfusion channel using a multichannel electronic pipettor. The plates were incubated elevated at a 70° angle at 37 °C, 5% CO_2_ to allow HMEC-1s and THP-1s to attach to the iHep/collagen in the gel channel above the phase guide. After 15 min incubation, 50 µL iHep maintenance media was added into medium inlets and outlets of the perfusion channel and the plates were incubated elevated at a 70° angle at 37 °C, 5% CO_2_ for an additional 45 min. The plates were then placed on the perfusion rocker platform (Mimetas, Leiden, Holland) set to cycle every 4 min to a maximum angle of approximately 15° to induce gravity-driven media to flow through the perfusion channel. The media were collected and exchanged every 1–2 days by aspirating and replacing media from medium inlets and outlets (50 µL in each).

For evaluation of drug metabolism, iHep co-cultured with THP-1/HMEC in the OrganoPlate^®^ 2-lane 96 were exposed on days 8 and 12 of culture to either 1 or 5 µM mixture of 20 pesticides. After chemical exposure, media were collected after 48 h and replaced with fresh iHep maintenance media. Separate wells were exposed to chemical mixture on either day 8 or 12 to avoid repeated exposures to the same cells. Each experimental condition was replicated three times.

### 2.6. iHep 2D Sandwich Culture and Chemical Treatments

For the iHep 2D sandwich model, differentiated iHep clusters were collected as previously described and resuspended into 3.33 mg/mL collagen. The iHep/collagen suspension (50 μL/well) was pipetted into wells on the 96-well plate. The plate was placed at 37 °C, 5% CO_2_ for 15 min to allow polymerization of the type 1 collagen gel. After that, 50 μL iHep maintenance media was added into each well, and plates were incubated at 37 °C, 5% CO_2_. The media were exchanged every 1–2 days.

Chemical metabolism was evaluated by exposing cells on days 4 and 8 of culture to either a 1 or 5 µM mixture of pesticides. One hundred microliters of chemical stock solution (2 or 10 µM) were added to wells to achieve a final chemical concentration of 1 or 5 µM and cell density of 1 × 10^5^ cells/well. After chemical exposure, media were collected after 48 h and replaced with fresh iHep maintenance media. Separate wells were exposed to chemical mixture on either day 4 or 8 to avoid repeated exposures to the same cells. For negative control, cell-free wells were used to account for chemical stability in the device during cell culture and exposure periods. Each experimental condition was replicated three times.

### 2.7. Functional Assays

Cell culture media were collected after exposure to pesticide mixtures in Organoplate^®^ 96-well plate and 2D sandwich culture then analyzed for a variety of biomarkers. The ELISA assays for albumin (Cat No.: E88-129, Bethyl Laboratories, Montgomery, TX, USA) and lactate dehydrogenase (Cat No.: ab102526, Abcam, Cambridge, UK) were performed using the manufacturer’s instructions.

### 2.8. LC-MS/MS Analyses

Sample extraction procedures and chromatographic conditions were previously reported in [34]. In brief, each sample (50 µL) was spiked with 10 µL of 10 µM internal standards, mixed with 100 µL of chilled acetonitrile, and then centrifuged at 10,000× *g* for 5 min. The supernatant was dried under vacuum using SpeedVac (Savant SPD1010, Beckman Coulter, Brea, CA, USA) and reconstituted with 50 µL of aqueous mobile phase prior to analyses. LC-MS/MS analysis was performed using 1290 Infinity II LC and 6470 triple quadrupole mass spectrometer (both instruments from Agilent Technologies, Santa Clara, CA, USA). Sample extract (10 µL) was chromatographed on a ZORBAX SSHD Eclipse Plus C18 column (3.0 × 50 mm, 1.8 µm, Cat No.: 959757-302; Agilent Technologies) with a guard column (2.1 × 5 mm, 1.8 µm, Cat No.: 821725-901; Agilent Technologies), and ionized using electrospray ionization. Analytical response was acquired in both positive and negative modes.

For positive ion compounds, mobile phases consisted of 0.1% formic acid in water (A) and 0.1% formic acid in methanol (B) using the following gradient: 2% B held for 1 min, B increased to 80% by 3 min, B increased to 95% by 4 min, B decreased to 2% by 5 min and held for 3 min for a total run time of 8 min per sample at a flow rate of 0.4 mL/min. For negative ion compounds, the LC gradient and flow rate were the same as in positive mode, except that mobile phase A was water and mobile phase B was acetonitrile.

### 2.9. GC-MS/MS Analyses

Fifty microliters of media sample were spiked with 10 µL of 10 µM internal standards, mixed with 50 µL of methanol and 200 µL of pentane: diethyl ether (1:1 *v*/*v*), vortexed briefly, and then centrifuged at 600× *g* for 5 min. Organic layer supernatants were transferred to a 2 mL amber vial and concentrated under nitrogen prior to GC analysis. Detection of analytes was achieved using a 7890B GC and 7010B triple quadrupole mass spectrometer (both from Agilent Technologies). Samples were injected (1 µL) in splitless mode. Analytes were separated with a VF-5ms GC column (60 m × 250 µm × 0.25 µm, Cat No.: CP8960; Agilent Technologies) and ionized using electron ionization. The column head pressure was set at 21.5 psi (148,237 Pa) with a constant flow rate at 1.2 mL/min using helium gas. Initial column temperature was held at 70 °C for 5 min, increased to 150 °C at 50 °C/min, ramped to 280 °C at 4 °C/min, and then held for 15 min. The total run time was 42.1 min. The injector temperature was set at 250 °C. The ion source and auxiliary transfer line temperatures were 300 °C. Electron multiplier voltage was set at 1884 V. Ultra-high purity nitrogen gas was used as the collision gas for all MS/MS experiments, and collision gas pressure was set at 16.8 psi (115,832 Pa).

### 2.10. IMS-MS Analyses

All nontargeted analyses were performed using a 6560 IMS-QTOF MS (Agilent Technologies) as detailed previously [30,36]. All individual standards for pesticide parent compounds (n = 20) and known metabolites (n = 10) were directly injected in triplicate into the electrospray ionization (ESI) source (positive and negative mode) and atmospheric pressure photo-ionization (APPI, negative mode only) to obtain collision cross section (CCS) and mass-to-charge ratio (*m*/*z*) values. Blanks were injected between standards to reduce the likelihood of carryover. For sample analyses, ESI was chosen as the optimal source to detect analytes in either positive or negative mode. During IMS-MS analyses, ions were passed through the inlet glass capillary, focused by a high-pressure ion funnel, and accumulated in an ion funnel trap. Next, ions were pulsed into the 78.24 cm-long IMS drift tube filled with nitrogen gas at a pressure of approximately 3.95 torr (527 Pa). Ions exiting the drift tube were refocused by a rear ion funnel prior to quadrupole time-of-flight (QTOF) MS detection. Detailed instrumental settings in ESI mode can be found in Table S1. Prior to instrumental analysis, the IMS-MS was tuned and a mass calibration was performed using Agilent Tune Mix from the manufacturer (Cat No.: G2421-60001, Agilent Technologies).

### 2.11. Determination of Intrinsic Clearance

In vitro hepatocyte clearance (Cl_in vitro_) of each chemical was estimated by substrate depletion approach assuming first-order kinetics for compound elimination [37]: Cl_in vitro_ = *k*V/N, where *k* = first-order elimination rate constant, V = incubation volume, and N = number of cells in the incubation. Cl_in vitro_ was further scaled up to the intrinsic hepatocyte clearance (Cl_int_) according to the equation [38]:

Cl_int_ = Cl_in vitro_ × HPGL × V*_l_* (1)

where HPGL = hepatocytes per gram liver (137 × 10^6^ cells/g) and V*_l_* = volume of the whole liver (1820 g).

### 2.12. Statistical Analyses

General descriptive statistical analyses were conducted using GraphPad Prism 9.0 (San Diego, CA, USA). Statistical significance (*p* < 0.05 was selected as a threshold) was tested with one-way ANOVA with Dunnett’s multiple comparisons test, or two-way ANOVA with Tukey’s multiple comparisons test as indicated in figure legends.

## 3. Results

The metabolism of pesticides in a mixture was evaluated through traditional (suspension and 2D sandwich cultures) and novel (OrganoPlate^®^ 2-lane 96) in vitro models using based on pluripotent stem cell-derived hepatocytes (iHep). The mixtures consisted of 20 pesticides where each individual compound was the same concentration (1 or 5 µM). Following chemical exposure, targeted and nontargeted analyses were performed (Figure 1). Additionally, hepatic biomarkers including albumin production and lactate dehydrogenase leakage were evaluated in 2D sandwich cultures and OrganoPlate^®^ 2-lane 96.

### 3.1. Liver Function Comparison between 2D Sandwich and OrganoPlate^®^ 2-Lane 96

**Figure 2:**
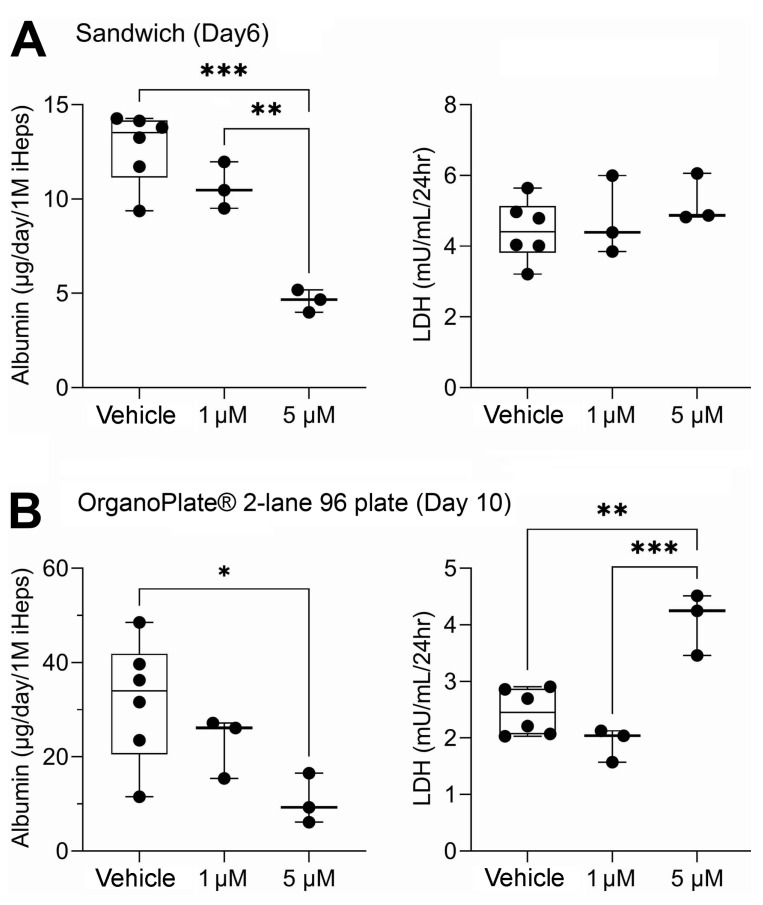
Albumin and lactate dehydrogenase (LDH) in traditional 2D sandwich culture (**A**) and OrganoPlate^®^ 2-lane 96 (**B**). Data are plotted as box (interquartile range) and whiskers (min–max range) with individual data points shown; horizontal line is median. One asterisk (*) denotes statistical differences at *p* < 0.05 (one-way ANOVA with Dunnett’s multiple comparisons test). Two asterisks denote differences at *p* < 0.01, and three asterisks denote statistical differences at *p* < 0.001.

iHep were pre-differentiated and then cultured for up to 10 days in 2D sandwich cultures and for up to 14 days in OrganoPlate^®^ 2-lane 96. Additionally, OrganoPlate^®^ 2-lane 96 included THP-1 monocyte-derived macrophages and HMEC-1 endothelial cells. Sandwich cultures and OrganoPlate^®^ 2-lane 96 each had two 48 h exposure periods. Sandwich cultures were exposed on days 4 and 8 of culture while OrganoPlate^®^ 2-lane 96 were exposed on days 8 and 12 of culture. Following the first exposure period in both models, media were collected and analyzed for hepatic biomarkers, albumin, and lactate dehydrogenase (Figure 2). Albumin production was lower in sandwich cultures (Figure 2A) as compared to OrganoPlate^®^ 2-lane 96 (Figure 2B). On average, albumin production in all three testing conditions (vehicle and two mixtures) was about 50% less in sandwich compared to OrganoPlate 2-lane 96. Overall, albumin production in both models was comparable to previous studies. In OrganoPlate 2-lane 96, iHep function was close to the lower range of albumin production levels in human liver [21] and higher than previously reported in this model [23]. For sandwich cultures, lower albumin production is expected [24]. Cell viability, as indicated by LDH release, was similar for vehicle and 1 µM mixture in sandwich cultures, but there was approximately a 12% decrease in live cells in the 5 µM mixture (Table S2). OrganoPlate^®^ 2-lane 96 exhibited a concentration-dependent decrease in cell viability with an average of 60% viability after exposure to the 5 µM mixture (Table S2). In both models, there was a significant decrease in albumin production in the 5 μM mixture conditions. Even though no increased LDH leakage was observed in sandwich cultures, a small, but significant, increase in LDH release was detected in OrganoPlate^®^ 2-lane 96. Thus, we concluded that due to some disruption in cell viability and functionality in the 5 μM condition, the data on chemical metabolism were most informative for the 1 μM experimental condition and subsequent figures present data from this arm of the study, and the other mixture data are presented in Figure S1.

### 3.2. Liver Metabolism Assessment through Targeted Mass Spectrometry Analyses

**Figure 3:**
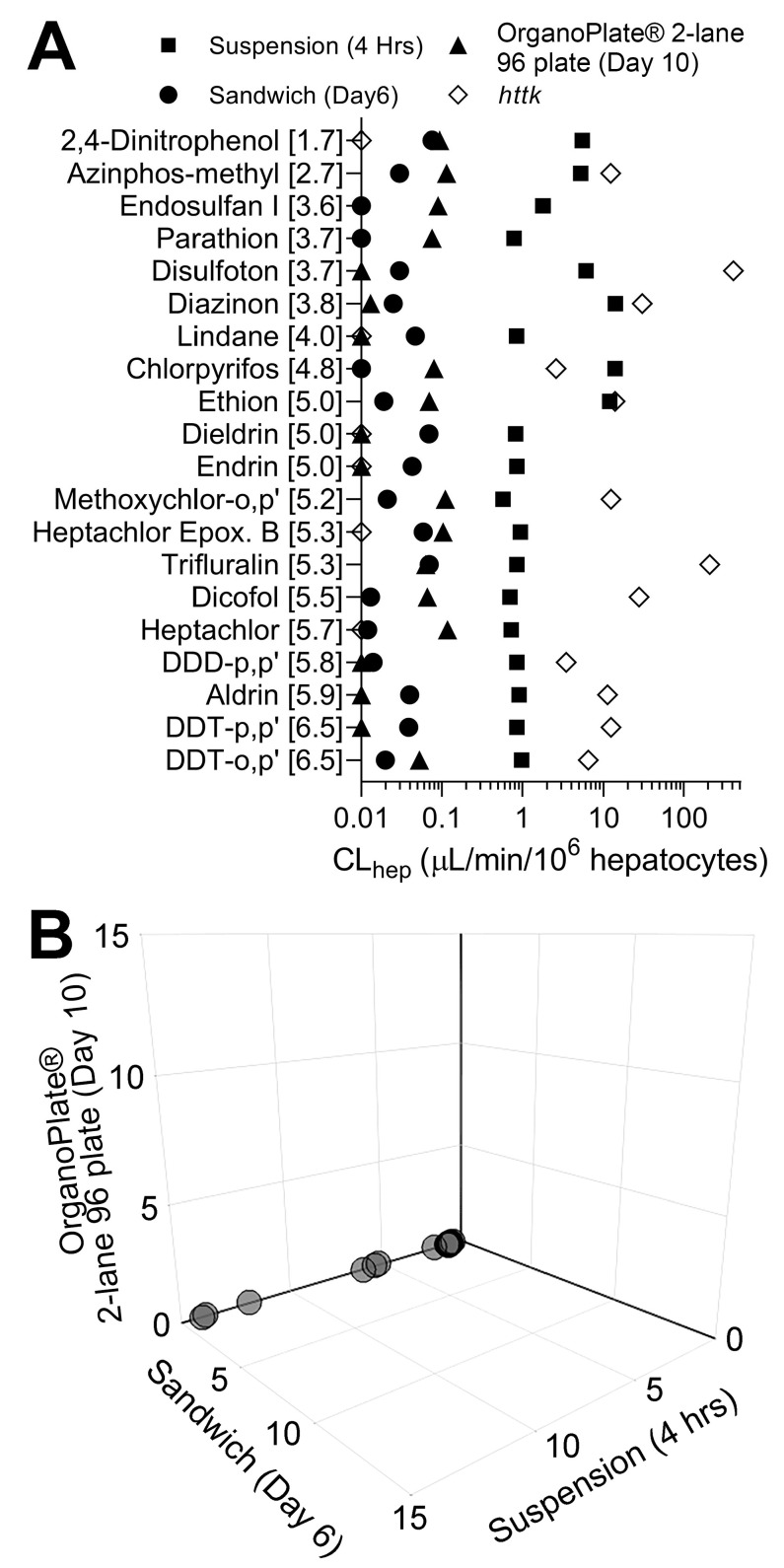
(**A**) In vitro hepatocyte clearance of chemicals in suspension culture (squares), sandwich culture (circles), OrganoPlate^®^ 2-lane 96 (triangles), and *httk* (diamonds). Plotted are in vitro hepatocyte clearances (mean) of each chemical listed (the number in brackets indicates the LogP of each compound). Experiments were conducted using induced pluripotent stem cell-derived hepatocytes (iHep). Each chemical was tested at 1 µM in a mixture setting. Clearance values reported for each chemical in *httk* R package version 1.10.1 were tested using primary human hepatocyte suspensions at 10 µM in a single chemical setting. (**B**) 3D plot showing hepatocyte clearance of each chemical across the three in vitro models used in this study. Axes represent in vitro clearance values for each compound in a 1 µM mixture. All data points for each model can be found in Table S3.

Following chemical treatments, media were collected in all three in vitro liver models and analyzed for the presence of all 20 pesticide parent compounds with targeted methods, which included liquid chromatography and gas chromatography coupled with tandem mass spectrometry (LC-MS/MS and GC-MS/MS). All 20 compounds in 1 µM equimolar mixtures were cleared at a rate of less than 1 µL/min/10^6^ hepatocytes in sandwich culture and OrganoPlate^®^ 2-lane 96 (Figure 3A). In suspension cultures, a majority of compounds were metabolized at approximately 1 µL/min/10^6^ hepatocytes. However, six compounds: 2,4-dinitrophenol, azinphos-methyl, disulfoton, diazinon, chlorpyrifos, and ethion were cleared at higher rates ranging from approximately 5 to 14 µL/min/10^6^ hepatocytes. Overall, compounds with a lower octanol–water partition coefficient (log P) showed higher clearance compared to the more lipophilic compounds in suspension.

In addition, Figure 3A plots clearance data reported in *httk* using suspension cultures of cryopreserved primary human hepatocytes; in those experiments, each chemical was tested individually at 10 µM. For 15 out of 18 compounds, *httk* data are far higher with respect to hepatocyte clearance values than those obtained in this study; it is unlikely that this difference is due to the use of iHep rather than primary human hepatocytes because we previously reported that hepatic clearance of these compounds in a mixture setting is generally far lower than that tested in single chemical experiments [34]. Next, we visualized a comparison of clearance rates for each chemical in 1 µM mixtures between in vitro models used in this study and a 3D plot, where axes represent each model tested (Figure 3B). Overall, this figure reveals a cluster of compounds that were cleared by iHep in suspension, but little metabolic clearance was observed in two other models (Figure 3B). The data for hepatic clearance using the 5 µM mixture are shown in Figure S1; however, these data shall be interpreted with caution because of the loss in functionality and lactate dehydrogenase leakage indicative of the loss of viability (Figure 2).

### 3.3. Comparison of In Vitro Hepatocyte Clearance Values Obtained from Targeted and Nontargeted Analyses

**Figure 4:**
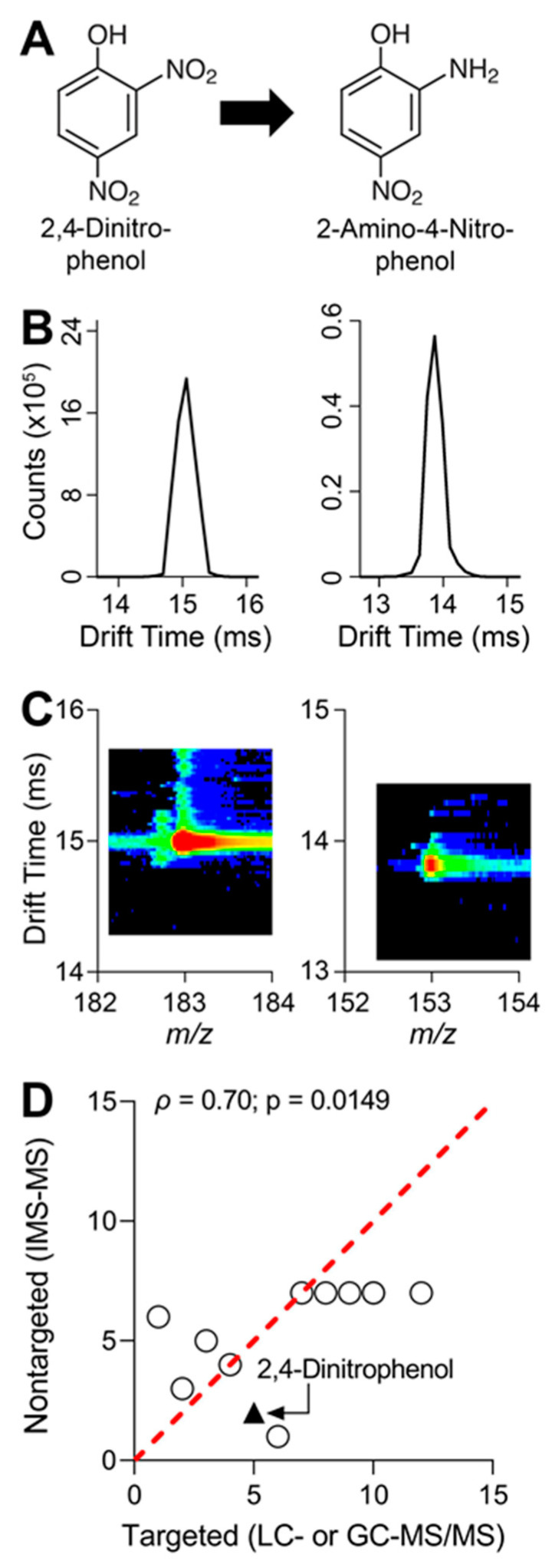
Metabolism pathway of 2,4-dinitrophenol (2,4-DNP), IMS-MS drift time chromatography of 2,4-DNP and major metabolite, and correlation of pesticides detected by targeted and nontargeted analyses: (**A**) Schematic of 2,4-DNP metabolism via sequential nitro group reduction to major metabolite, 2-amino-4-nitrophenol. (**B**) IMS-MS drift time vs. abundance (counts) chromatograms for 2,4-DNP (left panel) and 2-amino-4-nitrophenol (right panel). Data shown were generated by running analytical standards to obtain IMS drift time. (**C**) Plotted are IMS-MS drift time vs. mass-to-charge ratio spectra for 2,4-DNP (left panel) and 2-amino-4-nitrophenol (right panel). Drift time and mass-to-charge ratios were obtained by testing analytical standards. (**D**) Pair-wise ranked correlation (Spearman) plot of pesticides detected by targeted and nontargeted analyses (n = 12). For targeted and nontargeted analyses, each compound was ranked from highest (1) to lowest (12) in terms of in vitro clearance of 1 µM mixture in iHep suspension. 2,4-DNP is denoted by the black triangle. Spearman (ρ) correlation value is shown in the graph with corresponding *p*-value. Red dotted line is a unit line. All data values for targeted and nontargeted clearance and ranked correlation analysis can be found in Table S4.

Prior to starting cell cultures and subsequent chemical exposure, analytical standards for all 20 parent compounds and 10 metabolites were analyzed using IMS-MS through direct injection and with an electrospray ionization source. Following IMS-MS analyses, ions for all compounds were searched by using potential mass-to-charge ratios (*m*/*z*) from common forms of ionization including the addition/removal of protons, addition of sodium, or addition of ammonium. Twelve parent compounds and eight metabolites were detected and IMS collision cross section (CCS) values were collected to enhance feature matching (Table 2). From the list of twelve parent compounds and eight metabolites detected with IMS-MS, the conversion of 2,4-dinitrophenol to its major metabolite 2-amino-4-nitrophenol is shown as an example (Figure 4A). Direct injection of analytical standards in IMS-MS generates drift time peaks and a careful review of the peaks provides additional confidence in identifying each feature (Figure 4B). Additionally, spectra for *m*/*z* vs. IMS drift time are generated to further confirm the presence of each feature (Figure 4C).

Following chemical exposure, media were collected from each in vitro model, and then chemical extractions were performed. Targeted analyses revealed that a 1 µM mixture in the suspension of iHep showed the greatest metabolic capacity for a few select compounds (Figure 3). Following targeted analyses, IMS-MS was used for nontargeted screening. Our data revealed the presence of twelve parent compounds from each of the in vitro assays. Characterization of chemical metabolism with 1 µM mixture suspension was determined by calculating in vitro hepatocyte clearance for each parent compound. Correlation analysis revealed significant concordance between targeted and nontargeted methods (Figure 4D).

### 3.4. Metabolite Detection Using IMS-MS Nontargeted Analyses

**Figure 5:**
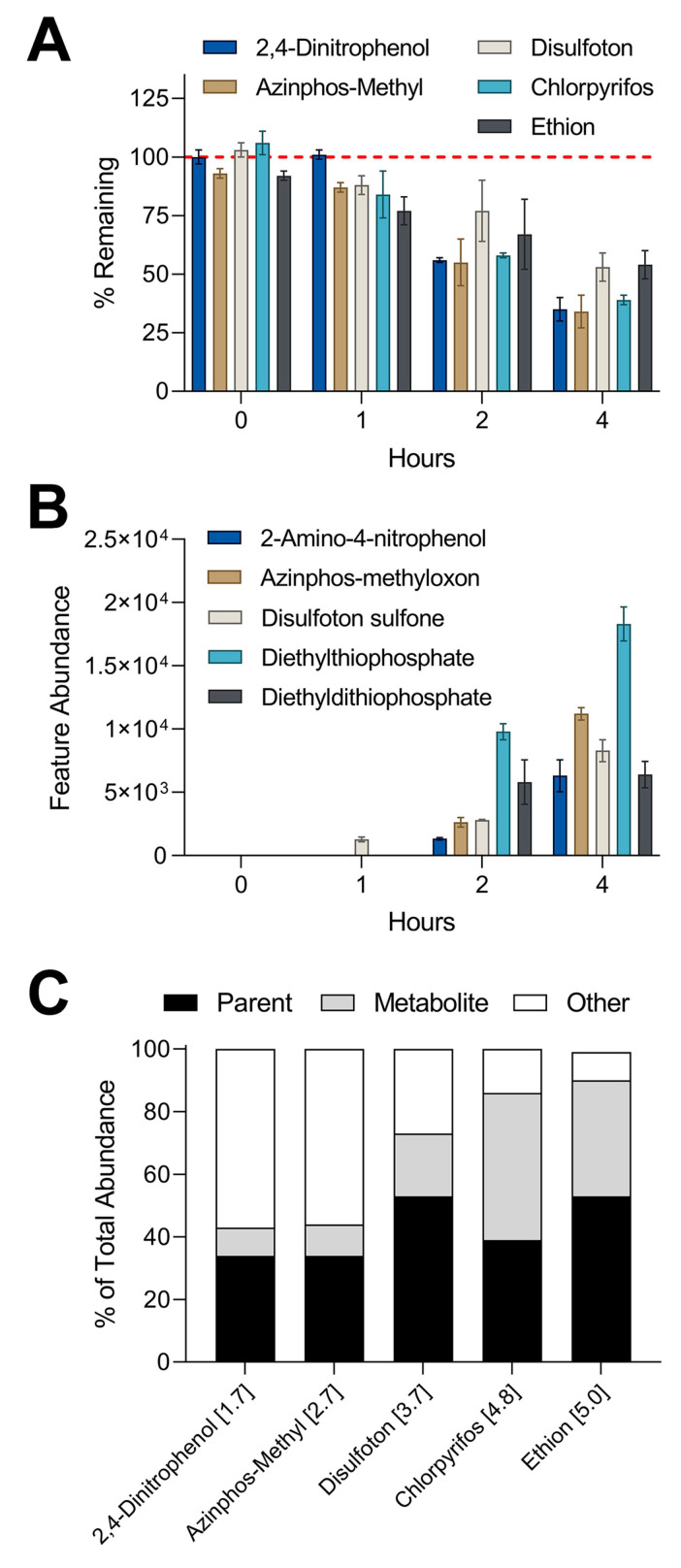
Comparison of in vitro metabolism of 1 µM equimolar mixture in suspension assay and metabolite formation through nontargeted analysis: (**A**) Plotted are % remaining values (mean ± SD) for chemicals that showed clearance in suspension assay. Values were derived by dividing the abundance of each chemical by the abundance of the corresponding compound in control experiment (boiled hepatocytes). (**B**) Graphed are abundances (mean ± SD) of metabolites detected through nontargeted analyses. Metabolites correspond to the parent compound by matching color. Two-way ANOVA with Tukey’s multiple comparisons tests revealed statistical differences between % remaining for parent compounds and feature abundances of metabolites. Summary of statistical information including *p* values can be found in Table S5. (**C**) Stacked bar graphs showing the mass balance distribution of abundances for each chemical between parent compound, metabolite, and other. The number in brackets next to each compound represents the log-*p* value.

The results from the targeted analyses showed that a 1 µM mixture in the suspension of iHep had the highest metabolic capacity among all in vitro models and test conditions. A subset of five compounds was chosen for nontargeted screening that previously showed high levels of metabolism in suspension culture: 2,4-dinitrophenol, azinphos-methyl, disulfoton, chlorpyrifos, and ethion. IMS-MS analyses revealed these five compounds displayed high levels of metabolism through nontargeted methods (Figure 5A), similar to the findings with targeted analyses (Figure 3). Furthermore, we used *m*/*z* and CCS values obtained from testing standards to screen for the presence of metabolites associated with the five parent compounds. IMS-MS analysis allowed the detection of five metabolites that were previously missed by targeted screening (Figure 5B). Our data indicated a time-dependent formation of each metabolite where the highest abundance for each feature occurred at the latest time point in the suspension assay. Lastly, we compared the abundance of each parent compound and associated metabolite to the abundance of the corresponding parent compound in our control experiments to estimate mass balance (Figure 5C). The percent of total abundance for parent compounds ranged from 34 to 53%. The metabolite percentage of total abundance varied from 9 to 47% while the remaining portion of “other” spanned from 9 to 57%. It is important to note that certain metabolites including diethylthiophosphate can be generated from more than one parent compound present in the chemical mixture [39,40].

## 4. Discussion

Understanding the metabolism and hepatic clearance of environmental chemicals is crucial to characterizing potential toxicities to human health. Traditional in vivo models can provide abundant metabolism information for xenobiotics [41,42]. However, there are major ethical and logistical concerns regarding the use of animals for toxicity studies, particularly given the number of chemicals that need evaluation [43,44]. To bypass these limitations, in vitro cultures of hepatocytes in various configurations and platforms can be used for rapid, high-throughput screening of xenobiotic compounds [45,46,47]. Two current in vitro liver models to assess metabolism include hepatocyte suspension and sandwich culture [18,48]. Suspension cultures offer several benefits such as being fairly high-throughput, retaining high levels of enzyme functionality (similar to in vivo), and typically yielding better estimates of clearance compared to monolayer cultures [17,49]. Nonetheless, suspension cultures also have limitations that include the loss of cell-to-cell interactions, short-term viability (4 h or less), and loss of cellular polarity [50,51]. The benefits of sandwich cultures include the restoration of in vivo hepatocyte polygonal morphology, prevention of decline in cell viability, and functional bile canaliculi [52,53]. However, there are disadvantages associated with long-term sandwich cultures such as loss of liver-specific functionality and decline in metabolic enzyme activity [54,55].

The shortcomings of traditional in vitro liver models have directly led to the development of novel 3D platforms known as liver microphysiological systems [56,57]. These devices utilize microfluidic technology to mimic the in vivo microenvironment of the liver, they often consist of microchannels that connect chambers to facilitate culture medium perfusion [58]. Additionally, these devices are designed for co-culture conditions, which allows investigators to add supporting cells that can enhance hepatocyte function [45,59]. As a result of improving hepatocyte function and phenotype, liver microphysiological models show great promise for in vitro studies of the liver metabolism and toxicity [60]. These improvements have shown that liver-centric devices can predict clearance, toxicity, and mechanism of action of certain pharmaceutical compounds to a considerable degree [21,61]. Although liver microphysiological systems have shown great success in predicting the kinetics of selective drugs, their utility in predicting the kinetics of environmental compounds remains largely unexplored [23,62,63]. Compared to pharmaceuticals, environmental compounds have a much wider range of physicochemical properties, which have a direct impact on biokinetics [64]. Furthermore, modeling real-life exposures to xenobiotics through more complex in vitro systems requires a focus on chemical mixtures [10,65,66]. Data generated from screening mixtures and characterizing metabolism are informative for extrapolation to potential in vivo effects.

In addition to studies of hepatic clearance, it is equally important to understand the formation of possibly injurious metabolites [67]. However, detecting metabolites from in vitro testing can be challenging because these compounds tend to be present at very low concentrations within small volumes [32]. Enhancements in analytical instrumentation, primarily in sensitivity and resolution of MS techniques, have provided the ability to simultaneously screen for hundreds, if not thousands of compounds present in a given sample [68]. These advances in technology have driven a shift from targeted to nontargeted analyses that can overcome sensitivity issues associated with chemical detection [31]. Furthermore, nontargeted analyses performed with high-resolution MS and separations coupled to MS, including IMS-MS have improved the characterization of metabolites following exposure to environmental contaminants [69,70].

In this study, we assessed the in vitro metabolism of a defined chemical mixture using iHep suspensions, sandwich cultures, and OrganoPlate^®^ 2-lane 96. Our data provide a direct comparison of metabolic capacities between traditional and novel in vitro systems. Additionally, we took advantage of the nontargeted analysis capabilities of IMS-MS to test a hypothesis that it will facilitate the detection of metabolites even if they are present at very low levels. Our results indicate that in vitro clearance was low for a majority of tested compounds across all three liver models. However, a subset of compounds did show measurable clearance in iHep suspensions, and through IMS-MS, we were able to detect the presence of five metabolites. From the subset of the five compounds that showed hepatocyte clearance in suspension culture, a majority of them were organophosphate insecticides. 2,4-Dinitrophenol is the only compound that is not in that class. 2-amino-4-nitrophenol has been previously identified as being the major metabolite of 2,4-dinitrophenol across multiple species [71]. The other four compounds (azinphos-methyl, disulfoton, chlorpyrifos, and ethion) can potentially yield similar metabolites based on previous studies in rodents and biomonitoring data available from humans exposed to organophosphates [72,73,74]. Out of the five metabolites that were detected in our study, diethylthiophosphate was formed at the highest amount. It is a known metabolite of chlorpyrifos and ethion, which can explain how it was formed at higher amounts compared to other metabolites that only have one compound contributing to their formation.

Various challenges remain for in vitro metabolite characterization using either targeted or nontargeted MS analyses. Targeted approaches provide absolute quantitation and are highly informative for in vitro-to-in vivo extrapolation; however, they are limited to only evaluating chemicals in their targeted lists. They are also unsuitable for quantifying a large number of chemicals due to differences in extraction and chromatography needs [75,76]. Many researchers have attempted to overcome these challenges by using multiple analytical platforms in parallel, in order to detect as many compounds as possible. For example, a previous study applied a combination of three different chromatographic modes for the analysis of plasma samples to detect metabolites associated with myocardial ischemia [77]. Nonetheless, the main disadvantage with this approach is an increase in cost associated with coupling various methods and analytical equipment.

A number of nontargeted analytical approaches have been proposed as a sensible path toward the characterization of metabolites and transformation products of environmental pollutants [78]. Nontargeted methods using IMS-MS are highly dependent on the ability to accurately identify metabolites through various data parameters that include *m/z* and CCS values [79]. Furthermore, data from nontargeted metabolomics are challenging to visualize and interpret based on the extensive amount of data generated [80]. However, current efforts have revealed major advantages with using technology such as IMS-MS for metabolite profiling that includes rapid screening time, ability to distinguish structural and stereo-isomers, and consistency in CCS values across different instrumental settings [81,82]. Improvements to the precision of these parameters are quickly evolving with the development of more standardized protocols for analysis and data interpretation [79,83,84]. As IMS-MS becomes more widely used and CCS data are populated in searchable metabolomic libraries, identifications using this knowledge base will continue to aid in increasing confidence for metabolite assignment.

Our study provides additional important clues with respect to the metabolic capacity of conventional and novel in vitro liver models for mixture clearance. Here, we illustrated that a majority of the 20 pesticides tested in a mixture did not exhibit appreciable clearance across the three platforms other than a handful of compounds in suspension culture. We found that testing equimolar mixtures at higher concentrations leads to a decrease in overall clearance in each model. This might be explained by a higher cumulative amount of pesticide exposure that leads to increases in hepatotoxicity that were statistically significant compared to vehicle control and lower testing concentration. Additionally, we compared our mixture data to single chemical exposure clearance data reported by *httk* (version 1.10.1) [85]. We observed much lower clearance values from mixture testing compared to individual chemicals, which is concordant with a previous study [34].

We note several limitations in our study. First, although iHep in sandwich cultures and OrganoPlate^®^ 2-lane 96 showed stability in terms of liver function, uncertainties remain about their fetal-like traits that can have a considerable impact on xenobiotic metabolism studies [86]. However, the use of pre-differentiated iHep in our in vitro models may be advantageous for future comparisons between studies because many use commercially available iHep from a single donor [87]. Second, our nontargeted IMS-MS analysis provided data that can be used to compare relative amounts of metabolites across models, but they do not provide absolute quantification of the features detected. Determining absolute concentrations would facilitate extrapolation efforts to estimate metabolite concentrations from in vivo exposures. Still, these nontargeted analyses provide guidance toward refining and performing more focused targeted screenings. Lastly, our designed mixture was created with each compound being present at the same concentration. However, real-life exposures to multiple xenobiotics do not occur at the same concentration. In fact, some exposures could occur at much lower levels, which would directly impact metabolic capacities. Nonetheless, future studies of in vitro mixture metabolism could incorporate in vivo exposure estimates to improve mixture compositions to better reflect real-life exposure scenarios.

In conclusion, we note that although the OrganoPlate^®^ 2-lane 96 model exhibited robust hepatic functionality in terms of high albumin production and low LDH leakage, its utility for investigating biokinetics may have limitations. The results of our study indicate that iHep suspension cultures maintain greater metabolic capacity compared to sandwich cultures and OrganoPlate^®^ 2-lane 96. For a majority of the 20 pesticides present in the tested mixtures, suspension cultures of iHep yielded intrinsic clearance rates that were 10 to 30-fold higher than sandwich culture and OrganoPlate^®^ 2-lane 96. Similarly, suspension culture was the only in vitro model that produced detectable metabolites. Therefore, among tested in vitro models, suspension cultures represent the most appropriate model for determining in vitro clearance and biotransformation of chemicals and mixtures. Furthermore, we conclude that IMS-MS is a useful analytical tool for nontargeted screening of xenobiotic transformation products and provides informative data needed for the comprehensive characterization of the toxicokinetics of chemicals and mixtures.

## Figures and Tables

**Table 1:** Test chemicals and metabolites analyzed in this study.

Chemical	CASRN	Vendor	Purity	Catalog No.
Test chemicals (Parent compounds)				
Aldrin	309-00-2	Chem Service	97.9%	N-11049
DDD-p,p’	72-54-8	Sigma-Aldrich	≥98%	35486
DDT-o,p’	789-02-6	Chem Service	99.5%	N-12708
DDT-p,p’	50-29-3	Sigma-Aldrich	≥98%	31041
Dicofol	115-32-2	Sigma-Aldrich	≥98%	36677
Dieldrin	60-57-1	Sigma-Aldrich	≥95%	33491
Endosulfan I	115-29-7	Sigma-Aldrich	≥98%	32015
Endrin	72-20-8	Sigma-Aldrich	≥98%	32014
Heptachlor epoxide B	1024-57-3	Chem Service	99.5%	N-12148
Heptachlor	76-44-8	Chem Service	98.6%	N-12147
Lindane	58-89-9	Sigma-Aldrich	≥96.5%	233390
Methoxychlor-o,p’	72-43-5	Sigma-Aldrich	≥98%	36161
Parathion	56-38-2	Chem Service	98.4%	N-12819
Trifluralin	1582-09-8	Sigma-Aldrich	≥98%	32061
2,4-Dinitrophenol	51-28-5	Sigma-Aldrich	≥98%	34334
Azinphos-methyl	86-50-0	Sigma-Aldrich	≥95%	45333
Chlorpyrifos	2921-88-2	Sigma-Aldrich	≥98%	45395
Diazinon	333-41-5	Sigma-Aldrich	≥98%	45428
Disulfoton	298-04-4	Sigma-Aldrich	≥98%	45460
Ethion	563-12-2	Sigma-Aldrich	≥95%	45477
Metabolites				
2-Amino-4-Nitrophenol	99-57-0	Sigma-Aldrich	96%	A70402
4-Amino-2-Nitrophenol	119-34-6	Sigma-Aldrich	≥95%	45946
Azinphos-methyl oxon	961-22-8	TRC	≥95%	G855650
DDA-p,p’	5359-38-6	Sigma-Aldrich	98%	100870
DDE-p,p’	72-55-9	Chem Service	99.3%	N-10875
Diazoxon	962-58-3	TRC	≥95%	D416890
Diethylthiophosphate	5871-17-0	Sigma-Aldrich	98%	445177
Diethyldithiophosphate	298-06-6	Sigma-Aldrich	90%	D93600
Dimethylthiophosphate	1112-38-5	TRC	≥95%	D495418
Disulfoton sulfone	2497-06-5	Sigma-Aldrich	≥95%	45871
Internal Standards				
Atrazine	1912-24-9	Sigma-Aldrich	≥98%	45330
Benzo[a]anthracene	56-55-3	Sigma-Aldrich	≥98.5%	B2209
Terbutryn	886-50-0	Sigma-Aldrich	≥98%	45677
Mifepristone	84371-65-3	Selleck Chem	>99%	S2606
Troglitazone	97322-87-7	Sigma-Aldrich	≥98%	T2573

**Table 2:** Chemicals detected with IMS-MS.

Chemical	Parent Compound(s)	*m*/*z*	CCS
2,4-Dinitrophenol	-	183.01	127.81
Azinphos-methyl	-	339.99	169.57
Disulfoton	-	297.02	165.2
Chlorpyrifos	-	371.91	172.35
Ethion	-	406.98	180.81
Heptachlor epoxide B	-	386.82	177.49
Trifluralin	-	336.12	161.69
Diazinon	-	327.09	174.89
Endosulfan I	-	404.82	175.19
Dieldrin	-	378.88	160.76
Aldrin	-	361.88	157.56
DDD-p,p’	-	316.95	170.62
Metabolites			
2-Amino-4-nitrophenol	2,4-Dinitrophenol	153.03	116.54
4-Amino-2-nitrophenol	2,4-dinitrophenol	153.03	117.87
Azinphos-methyl oxon	Azinphos-methyl	324.02	177.67
DDA-p,p’	DDD-p,p’	278.98	152.46
Diethylthiophosphate	Diazinon, Chlorpyrifos, Ethion	171.02	129.33
Diethyldithiophosphate	Ethion	187.00	135.08
Dimethylthiophosphate	Azinphos-methyl	142.99	113.21
Disulfoton sulfone	Disulfoton	307.03	156.79

## Data Availability

Data are available from the corresponding author upon request.

## Supplementary Materials

The following are available online at https://www.mdpi.com/article/10.3390/toxics10100566/s1, Figure S1: Hepatocyte clearance of 5 µM mixture, Table S1: IMS-MS Instrumental Parameters. Table S2: Cell Viability, Table S3: Figure 3 clearance values, Table S4: Figure 4 clearance and rank values, and Table S5: Figure 5 statistical analyses.

## Abbreviations

APPI, atmospheric pressure photo-ionization; ATSDR, Agency for Toxic Substances and Disease Registry; CCS, collision cross section; DMSO, dimethyl sulfoxide; ECM, extracellular matrix; ESI, electrospray ionization; httk, high-throughput toxicokinetics; iHep, induced pluripotent stem cell-derived hepatocytes; IMS-MS, ion mobility spectrometry–mass spectrometry; GC-MS/MS, gas chromatography tandem–mass spectrometry; LC-MS/MS, liquid chromatography tandem–mass spectrometry.
