# Supplementary figures and images for: Evaluation of Metabolism of a Defined Pesticide Mixture through Multiple In Vitro Liver Models
Source: Toxics. 2022 Sep 27;10(10):566. doi: 10.3390/toxics10100566 (PMC9609317; doi:10.3390/toxics10100566)

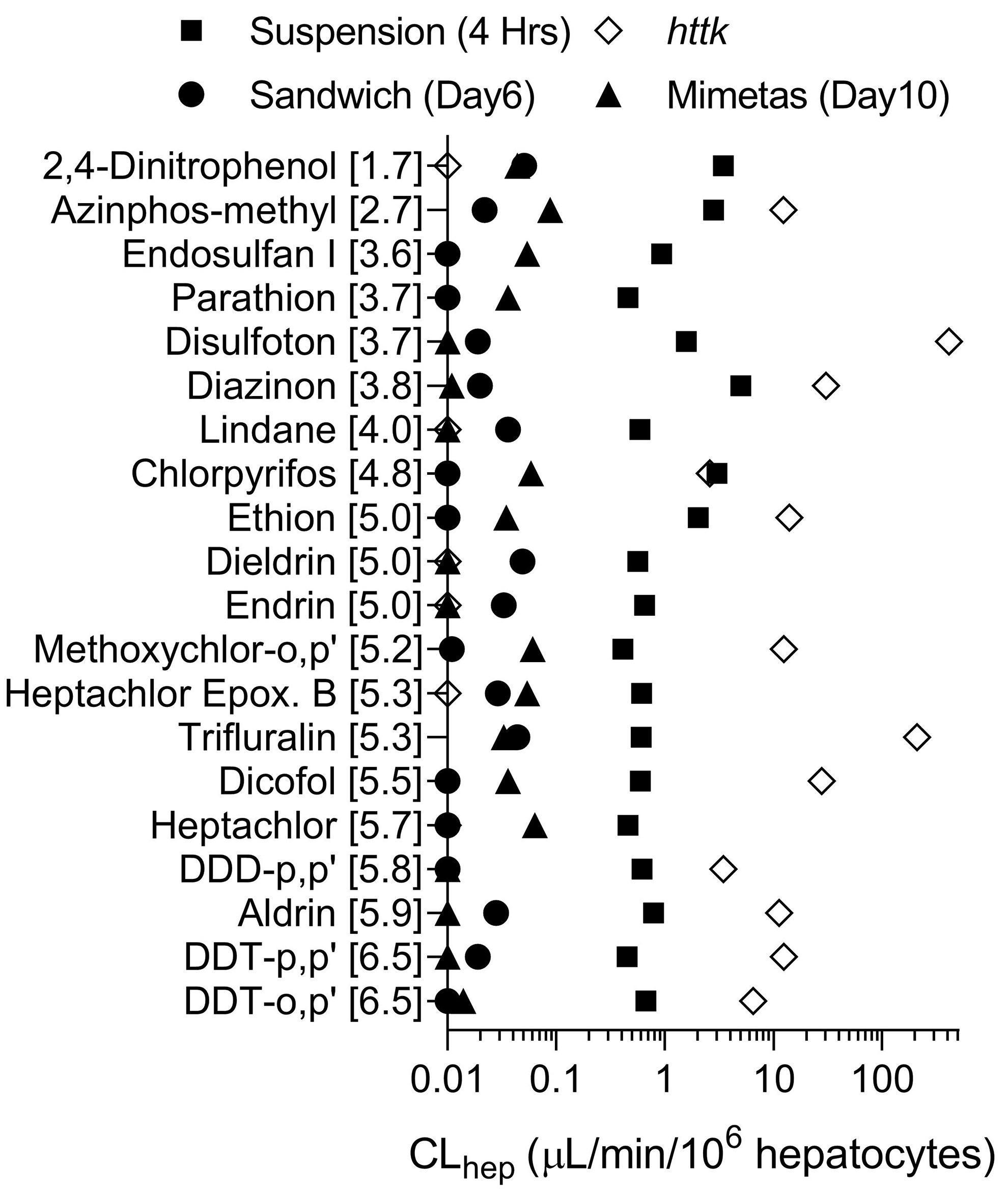

Supplement: Supplementary file 1 [file toxics-10-00566-s001.zip › Supplemental Figure S1.jpg]
